# Rapid and sustained T cell-based immunotherapy against invasive fungal disease via a combined two step procedure

**DOI:** 10.3389/fimmu.2023.988947

**Published:** 2023-04-05

**Authors:** Sabine Tischer-Zimmermann, Elisabeth Salzer, Tamires Bitencourt, Nelli Frank, Christine Hoffmann-Freimüller, Julia Stemberger, Britta Maecker-Kolhoff, Rainer Blasczyk, Volker Witt, Gerhard Fritsch, Wolfgang Paster, Thomas Lion, Britta Eiz-Vesper, René Geyeregger

**Affiliations:** ^1^ Institute of Transfusion Medicine and Transplant Engineering, Hannover Medical School, Hannover, Germany; ^2^ St. Anna Children’s Cancer Research Institute (CCRI), Vienna, Austria; ^3^ Department of Pediatrics, St. Anna Children’s Hospital, Medical University of Vienna, Vienna, Austria; ^4^ Department of Pediatrics, Laboratory for Pediatric Immunology, Willem-Alexander Children’s Hospital, Leiden University Medical Center, Leiden, Netherlands; ^5^ Department of Pediatric Hematology and Oncology, Hannover Medical School, Hannover, Germany; ^6^ German Center for Infection Research (DZIF), Partner Site Hannover-Braunschweig, Hannover, Germany; ^7^ Department of Pediatrics, Medical University of Vienna, Vienna, Austria

**Keywords:** HSCT, *Aspergillus* infections, T-cell immunotherapy, *Aspergillus*-specific T cells, T-cell expansion, cytokine secretion

## Abstract

**Introduction:**

*Aspergillus fumigatus* (Asp) infections constitute a major cause of morbidity and mortality in patients following allogeneic hematopoietic stem cell transplantation (HSCT). In the context of insufficient host immunity, antifungal drugs show only limited efficacy. Faster and increased T-cell reconstitution correlated with a favorable outcome and a cell-based therapy approach strongly indicated successful clearance of fungal infections. Nevertheless, complex and cost- or time-intensive protocols hampered their implementation into clinical application.

**Methods:**

To facilitate the clinical-scale manufacturing process of *Aspergillus fumigatus*-specific T cells (ATCs) and to enable immediate (within 24 hours) and sustained (12 days later) treatment of patients with invasive aspergillosis (IA), we adapted and combined two complementary good manufacturing practice (GMP)-compliant approaches, i) the direct magnetic enrichment of Interferon-gamma (IFN-γ) secreting ATCs using the small-scale Cytokine Secretion Assay (CSA) and ii) a short-term *in vitro* T-cell culture expansion (STE), respectively. We further compared stimulation with two standardized and commercially available products: Asp-lysate and a pool of overlapping peptides derived from different Asp-proteins (PepMix).

**Results:**

For the fast CSA-based approach we detected IFN-γ^+^ ATCs after Asp-lysate- as well as PepMix-stimulation but with a significantly higher enrichment efficiency for stimulation with the Asp-lysate when compared to the PepMix. In contrast, the STE approach resulted in comparably high ATC expansion rates by using Asp-lysate or PepMix. Independent of the stimulus, predominantly CD4^+^ helper T cells with a central-memory phenotype were expanded while CD8^+^ T cells mainly showed an effector-memory phenotype. ATCs were highly functional and cytotoxic as determined by secretion of granzyme-B and IFN-γ.

**Discussion:**

For patients with IA, the immediate adoptive transfer of IFN-γ^+^ ATCs followed by the administration of short-term *in vitro* expanded ATCs from the same donor, might be a promising therapeutic option to improve the clinical outcome.

## Introduction

Invasive fungal diseases (IFDs) including *Aspergillus fumigatus* (Asp) and Candida constitute a major cause of morbidity and mortality in immunocompromised patients after allogeneic hematopoietic stem cell transplantation (HSCT) (1). Various risk factors, such as ongoing neutropenia, graft versus host disease (GvHD), the applied conditioning regimen, indwelling catheters, treatment with complement-inhibitory antibodies, and delayed immune reconstitution, increase the susceptibility to serious fungal infections. The overall one-year cumulative incidence of IFDs in transplant patients varies from 3.4 to 12% depending on several factors such as HLA-mismatch, induction, or consolidation therapy for acute leukemia (2, 3). Of all possible IFDs, invasive aspergillosis (IA) was the most common, followed by invasive candidiasis (2). The mortality rates of patients with IA differ between 25.4 (2, 4) and 67.7% (5). Despite these high variations, mortality rates have increased over the last years (5). Antifungal drugs such as Voriconazole, Amphotericin B in conventional or liposomal formulation, and Posaconazole showed only limited efficacy, especially against certain strains, and are associated with severe side effects (6–8).

Apart from the complex interaction of different innate immune cells, such as neutrophils, monocytes/macrophages (9), and natural killer (NK) cells (10), also specific CD4^+^ T helper (Th) cells play a crucial role in the adaptive host defense against Aspergillus infections (11, 12). In line with the importance of the adoptive immunity, the recovery from IA was correlated with the induction of CD4^+^ Asp-specific T cells (ATCs) (13), which was similar to what was found for virus-specific T cells (VSTs) in patients with adenovirus (AdV) infection (14, 15).

Within the last decade, several clinical trials demonstrated the adoptive transfer of VSTs as a safe and effective therapeutic strategy in immunocompromised patients post HSCT (16). For clinical applications, most T-cell products are manufactured by either immunomagnetic separation after short antigen stimulation *via* the good manufacturing practices- (GMP-) compliant large-scale CliniMACS cytokine capture system (IFN-gamma) (IFN-γ CCS) using the fully automated CliniMACS Prodigy device (17) or *ex vivo* expanded *via* long-term (4-6 weeks) (18) or faster and less cost-intensive short-term (9-12 days) expansion (STE) protocols (19–21). In contrast to studies showing successful adoptive transfers of VSTs, so far only one clinical trial has been performed on the infusion of ATCs (22). In this study, 35 patients, who were treated with ATCs (selected *via* limited dilution), cleared IA more often than did patients in a control group (46 patients). ATCs could be detected within three weeks after infusion and remained stable over time (22). Since then, only the study by Gottlieb et al. has investigated and evaluated the safety and efficacy of multi-pathogen-specific T cells as prophylactic treatment against viral and fungal infections/reactivations including Aspergillus in 12 patients following HSCT (23). Within 30 days of infusion, an increase in antigen-specific activated CD8^+^ T cells was observed, with no acute infusion-related toxicity and no infection-related death in any of the patients. Currently, two other phase I/II clinical trials based on either the adoptive transfer of Aspergillus Th1 cells for patients with proven IA after HSCT (EudraCT: 2013-002914-11) or third-party donor-derived specific T cells against Aspergillus and Candida species are still ongoing (clinicaltrial.gov: NCT02779439). Encouraged by this first clinical trial, different methods, mainly based on immunomagnetic selection followed by an extensive *ex vivo* expansion period (to increase cell numbers), have been developed. For these procedures, different stimuli such as lysates (24–27) or a limited choice of different defined overlapping peptide pools covering the sequence of Aspergillus proteins f16 (28), Crf1 (29), or a combination of Crf, Gel1 and Pmp20 (30), have been used. However, the rather highly complex and cost -intensive protocols hampered their implementation into clinical applications. In the meantime, as mentioned above, mainly two protocols, the IFN-γ CCS and STE, have emerged as “gold standards” - at least for the clinical application of VSTs (17, 19, 31, 32). Currently, a GMP-grade Asp-lysate and further promising peptide pools, e.g. f22, PMD SHOT etc. (33), are available, allowing the extension and adaptation of current methods to GMP-compliant procedures. The first adaptation of a virus-specific- to an Asp-specific STE-based protocol was recently performed by Papadoupoulou et al. (34) Peripheral blood mononuclear cells (PBMCs) were isolated from healthy donors for T-cell expansion over 9-11 days with either a GMP-grade Asp-lysate or a mixture of three different peptide pools (Crf1, Gel1 and SHMT) and a combination of interleukin- (IL-) 4 and IL-7. However, the method chosen always depends on various parameters, such as the patient´s health status, the availability of a suitable donor, the number of ATCs required, the costs and the regulatory requirements in different countries. Some studies showed that, similar to e.g. AdV (35), patients could die within few days after diagnosis of IA (36). Nevertheless, the fastest method so far - the IFN-γ CCS – results in limited numbers of antigen-specific T-cells in the final cell product after the immunomagnetic selection process. The STE approach, on the other hand, takes at least 2-3 weeks, including quality controls before product release.

In this study, we investigated whether the two main clinically established strategies i) the IFN-γ CCS and ii) the STE could be adapted and combined to enable rapid and sufficient generation of clinically applicable ATCs against the Asp-lysate and overlapping peptide pools from different Asp-proteins.

## Methods

### Study population

Peripheral blood was obtained from healthy thrombocyte donors and volunteers, who had been tested positive for the presence of ATCs *via* IFN-γ enzyme-linked immunospot (EliSpot) assay. All donors belong to the alloCELL registry (www.allocell.org) of Hannover Medical School and were pretested for cytomegalovirus (CMV) and Epstein-Barr virus (EBV) serology as described previously using commercially available IgG Western blot kits (recomLine, Mikrogen, Neuried, Germany) (37, 38). The study population consists of 102 female and 218 male donors (n=320), being 19.1 to 69.6 (Median = 43.1) years old (**Table 1**). Of the donors, 47.2% were CMV seropositive and 96.4% EBV seropositive, and all donors tested had no acute infection at the time of donation. The study was approved by the local Ethics Committee (EK Nr. 514/2011, 2519-2014, 2744-2015, 3331-2016). Peripheral blood mononuclear cells (PBMCs) were isolated by standard density gradient separation in Hannover, one part was directly used for small-scale IFN-γ cytokine secretion assay (CSA), the other one was sent to Vienna to prepare the short-term *in vitro* expansion (STE).

**Table 1:** Description of the healthy donor cohort.

Donor Cohort (n=320)			
Gender	Age	Serostatus	
(n, female/male)	(median, range) [years]	CMV (% pos, +/-)	EBV (% pos, +/-)
102/218	43.1 (19.1 – 69.6)	47.2 (151/169)	96.4 (239/9)

CMV serostatus was tested for all donors and 248/320 donors were tested for EBV, Epstein-Barr virus; CMV, cytomegalovirus.

Shown are absolute numbers (gender distribution), median and range (age in years) or proportion and absolute numbers (CMV- and EBV-serostatus).

### Monitoring of ATCs *via* enzyme-linked immunospot assay

**Figure 1:**
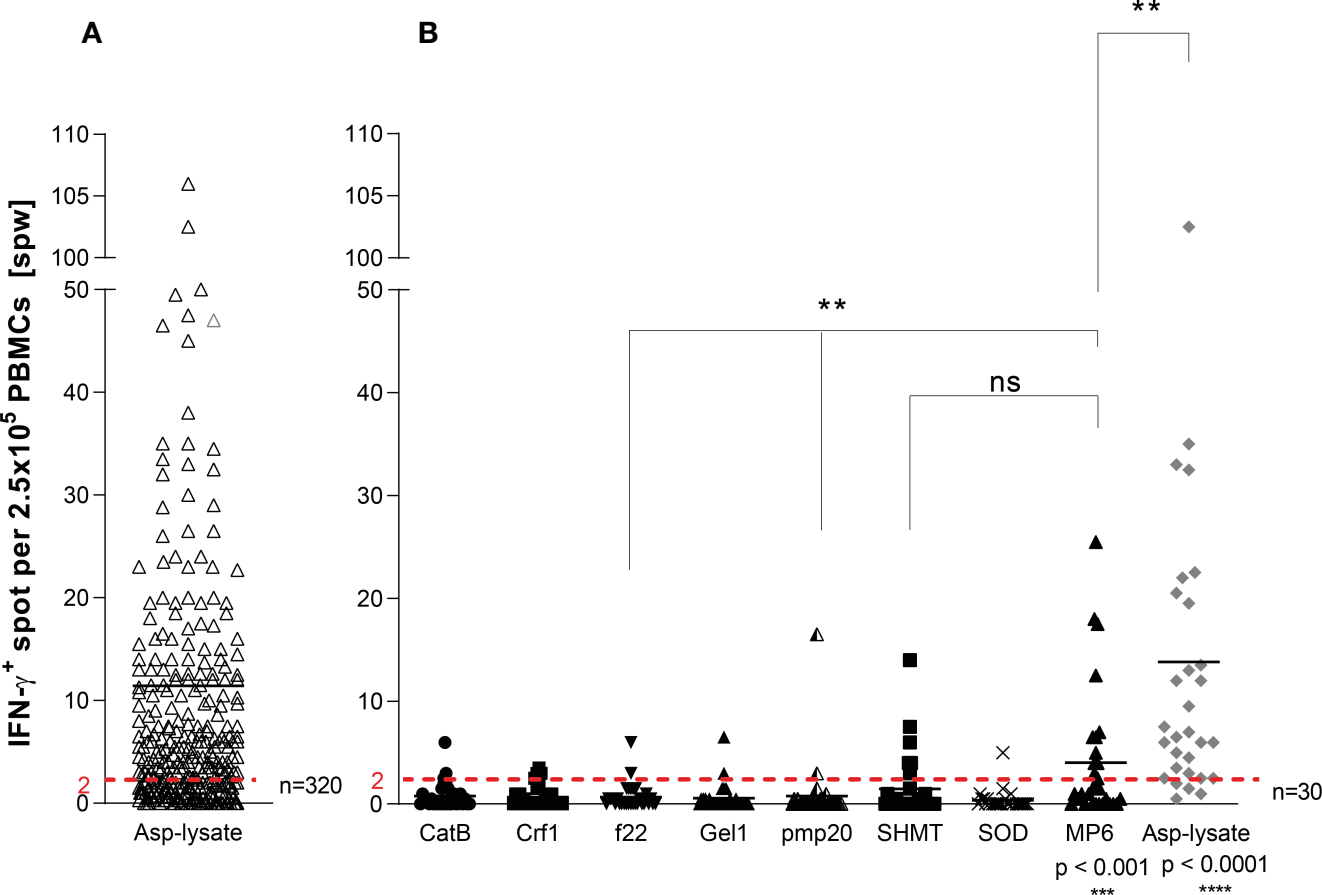
Screening for Asp-specific T cells by IFN-γ EliSpot. ATC responses elicited by the **(A)** *Aspergillus fumigatus* (Asp) lysate alone (n=320) or with **(B)** Apsp-lysate (n=30/320) and overlapping peptide pools from Aspergillus (CatB, Crf1, f22, Gel1, pmp20, SHMT, SOD) and *Candida albicans* (MP65) (n=30) were determined by IFN-γ EliSpot assays. 2.5 x 10^5^ cells/well of isolated PBMCs from healthy donors were stimulated with the investigated peptide pools (1µg per peptide/ml) and the Asp-lysate (50 µg/ml) for 16 hours on anti-IFN-γ precoated EliSpot plates. Results are indicated as number of spots per well (spw) after the subtraction of background spots (negative control = unstimulated cells). The cut-off value for a positive antigen-specific T-cell response was defined with ≥ 2 antigen-induced spw. Data are shown in total as individual result and mean. Statistically significant difference between MP6 or Asp-lysate to the other peptide pools, unless otherwise highlighted, is indicated by (**p < 0.01,***p<0.001 and ****p<0.0001). ns, not significant.

**Figure 2:**
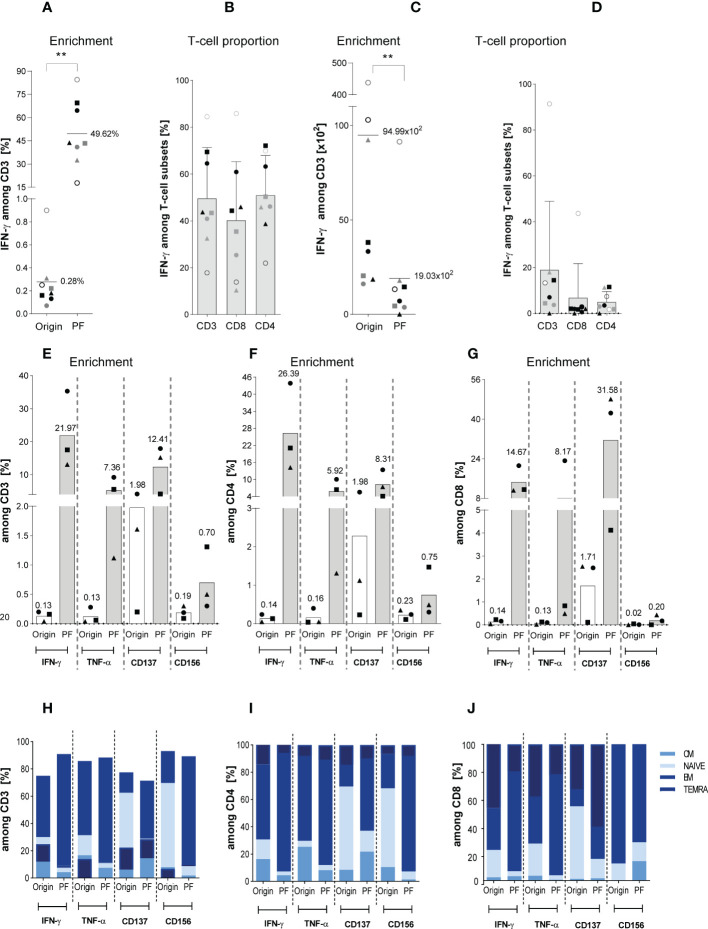
Characterization of magnetically selected ATCs. The enrichment efficiency of *in vitro* stimulated Asp-specific T cells was assessed by magnetic selection using either the IFN-γ cytokine secretion assay (CSA) or Microbeads kits in response to IFN-γ, TNF-α, CD137, and CD156 as specific T-cell activation marker. 1 x 10^7^ isolated PBMCs were stimulated overnight with the Asp-lysate (50 µg/ml) while unstimulated PBMCs served as the negative control. Aliquots of the respective cell fractions collected before (original fraction, Origin) and after enrichment (positive fraction, PF) were used for detailed characterization of activated T-cell subsets by multicolor flow cytometry. **(A)** Frequency of IFN-γ^+^ Asp-specific CD3^+^ T cells before (Origin) and after magnetic selection (PF). **(B)** Relative frequency of IFN-γ^+^ Asp-specific T-cell subsets (CD3, CD8 and CD4) within the PF after magnetic enrichment. **(C)** Total cell numbers of CD3^+^/IFN-γ^+^ Asp-specific T cells contained in the Origin and PF. **(D)** Total cell numbers of IFN-γ^+^ Asp-specific T-cell subsets (CD3^+^, CD8^+^ and CD4^+^) within the PF. Results are displayed as individual results (n=8) and mean ± SD. The different symbols represent the individuals in Figures 5A–D. Statistically significant difference is indicated by (**p < 0.01). **(E, F)** Frequency of Asp-specific (E) CD3^+^, (F) CD4^+^, and **(G)** CD8^+^ T cells before (Origin) and after magnetic selection (PF) using IFN-γ, TNF-α, CD137, and CD156 as specific selection marker. Findings are displayed as individual results (n=3) and as the mean percentage of Asp-specific T cells. **(H-J)** Phenotypic analyses regarding naïve (N), central memory (CM), effector memory (EM) and terminally differentiated effector memory (TEMRA) CD3^+^, CD4^+^ and CD8^+^ T cells were performed for the IFN-γ^+^, TNF-α^+^, CD137^+^, and CD156^+^ T cells of both fractions (Origin, PF) and are visualized by the respective mean frequencies (n=3). The healthy individuals used belonged to our cohort of donors (n=320) who were pre-tested for the presence of Asp-specific T cells.

Detection of IFN-γ-producing ATCs was achieved by IFN-γ EliSpot assay as previously described (39). Briefly, PBMCs were seeded on 24-well plates at a concentration of 1 x 10^7^ cells/ml. After overnight resting, PBMCs were co-cultured in anti-IFN-γ pre-coated EliSpot plates (Lophius Biosciences, Regensburg, Germany) for 16 hours at a density of 1 x 10^5^ (**Figure 1**) and 2.5 x 10^5^ (**Figure 2**) cells/well in the presence of the investigated overlapping peptide pools derived from *Aspergillus fumigatus*- (CatB1, Crf1, f22, Gel1, pmp20, SHMT, SOD) and *Candida albicans* (MP65) and Asp-lysate (all from Miltenyi Biotec, Bergisch Gladbach, Germany). Each peptide pool consists of peptides of 15 amino acid length with 11 amino acid overlap covering the complete protein sequence, which were used at a final concentration of 1 µg per peptide/ml and Asp-lysate was used at a final concentration of 50 µg/ml. PBMCs cultured with medium alone (RPMI1640, Lonza, Vervies, Belgium) with 10% human AB serum (C.C.pro, Oberdorla, Germany) or in the presence of 1 µg/ml staphylococcal enterotoxin B (SEB, Sigma-Aldrich, Hamburg, Germany) served as negative and positive controls, respectively. Spots of IFN-γ^+^ cells were counted and analyzed using the AID EliSpot spectrum reader system (AID, Strassberg, Germany) and the results were expressed as the number of spots per well (spw). The mean number of spots in the negative control was subtracted from the mean number of spots in the antigen wells. The cut-off value for a positive response was defined at ≥ 2 spw.

### Magnetic enrichment and phenotypic characterization of activated ATCs

The enrichment efficiency of *in vitro* stimulated ATCs was assessed by using IFN-γ, TNF-α, CD137 and CD154, respectively as specific T-cell activation marker. IFN-γ CSA and MircoBeads kits for labelling CD137-, CD154-, TNF-α-expressing cells were used according to the manufacturer’s instructions (all from Miltenyi Biotec). Briefly, after overnight resting, 1 x 10^7^ isolated PBMCs were stimulated with the Asp-lysate (50 µg/ml) or PepMix (consisting of the Asp- and *C. albicans*-derived peptides pools CatB, Crf1, f22, Gel1, pmp20, SHMT, SOD, MP65; 1 µg of each peptide/ml) for 16 hours. Unstimulated PBMCs served as the negative control. Activated T cells were specifically captured during the magnetic cell sorting enrichment process by using antibodies against TNF-α, IFN-γ, CD137, CD154, and paramagnetic anti-fluorochrome antibody-conjugated microbeads. Aliquots of the respective cell fractions collected before (original fraction, Origin) and after enrichment (positive fraction, PF) and were used for detailed phenotypic characterization of activated T-cell subsets by multicolor flow cytometry using the FACSCanto 10c system and FACSDiva Software version 8.0.1 (BD Biosciences, Heidelberg, Germany). The distribution of viable and dead cells in these fractions was analyzed by 7AAD (7-amino-actinomycin D) staining (BD Biosciences). In addition to anti-IFN-γ phycoerythrin (PE), anti-TNF-α allophycocyanin (APC), anti-CD137 APC, and CD154 APC (all from Miltenyi Biotec), cells were stained with anti-CD45 allophycocyanin plus cyanin-7 (APC-Cy7), anti-CD3 fluorescein (FITC), anti-CD4 Alexa Fluor 700, anti-CD45RA BV510, and anti-CD62L BV421 mAbs (all from BD Biosciences) according to the manufacturer’s protocols.

### Short-term-expansion of Asp-specific T cells

For short-term expansion (STE), 2 x 10^7^ PBMCs were cultured in G-REX devices (Wilson Wolf Manufacturing, New Brighton, MN) in 2 ml AIM-V medium (Invitrogen, Carlsbad, CA) supplemented with 25 mmol/l L-Glutamin (ThermoFisher Scientific, Waltham. MA), 25 mmol/l HEPES (ThermoFisher Scientific) and 2% Octaplas AB (Octapharma, Vienna, Austria) (named AIMV^+ + +^, as already described (19)) and stimulated for 12 days with either the Asp-lysate (50 µg/ml) alone or with combined overlapping peptide pools (referred to as PepMix) from Asp and *Candida albicans* (CatB1, Crf1, f22, Gel1, pmp20, SHMT, SOD, MP65; all from Miltenyi Biotec; 0.6 nmol/ml). On day six, cells were washed with AIMV^+ + +^ and resuspended and restimulated as on day 0. On day nine, IL-15 (CellGenix, Freiburg, Germany, 5 ng/ml) was added to the cell culture and seATCs were characterized by multi-parameter flow cytometry and IFN-γ EliSpot on day 12.

### Detection of seATCs by IFN-γ EliSpot assay

PBMCs (3 x 10^5^) or Asp-lysate- as well as PepMix-expanded seATCs (0.5 x10^5^) were restimulated for 16 hours with the appropriate antigen (Asp-lysate, single peptide pools or PepMix, as mentioned above) in triplicates. The secretion of IFN-γ was measured on an EliSpot device from C.T.L (Immospot^®^ S5 Versa, Bonn) using the human IFN-γ EliSpot kit (C.T.L) according to the manufacturer’s instructions and analyzed with the Immunospot software 5.0 (C.T.L). The limit of detection and release criterion was 2 spw per 10^5^ cells for PBMCs (as mentioned above) and 80 spw/10^5^ cells for seATCs after subtraction of background counts (unstimulated cells), respectively.

### Intracellular cytokine staining (ICS) of seATCs

For the intracellular cytokine staining (ICS), 2 to 2.5 x 10^5^ ATCs were incubated with autologous CD3-depleted PBMCs (CD3 stained and sorted *via* the BD FACSAria) at a effector to target (E:T) ratio of 3:1 or 2:1 or 1:1 in 200 µl AIMV^+ + +^ in a 96-well round bottom plate (Sarstedt, Nümbrecht, Germany). Cells were either unstimulated (negative control) or stimulated for 16 hours with the Asp-lysate (50 µg/ml, Miltenyi Biotec), single peptide pools, PepMix (Miltenyi Biotec) at a final concentration of 1 µg per peptide/ml or with SEB (1 µg/ml, Sigma Aldrich; as positive control). In addition to the stimuli, GolgiStop and GolgiPlug (both BD Biosciences) for protein transport inhibition were added as recommended by the manufacturer’s instructions. After incubation, cells were washed once with PBS (Lonza, Basel, Switzerland) and stained for 30 minutes at 4°C with fixable LIVE/DEAD™ Aqua Dead Cell Stain Kit (eBioscience/Thermo Fisher Scientific, Waltham. MA) to assess cell viability. After a washing step, cells were resuspended in 50 µl PBS and stained with the following surface antibodies for 20 minutes at 4°C: CD3 Phycoerythrin-CF594 (PE-CF594), CD8 APC, CD107a FITC, CD154 PE, CD137 BV650 (BD Biosciences), CD4 Peridinin-Chlorophyll-Protein (PerCP-eFluor710, eBioscience/Thermo Fisher Scientific). Cells were then resuspended in 150 µl permeabilization buffer and stained for IFN-γ Brilliant Violet 421 (BV421, BD Biosciences) and TNF-α PE-Cyanine7 (PE-Cy7, BD Biosciences) for 20 minutes at room temperature (RT). Samples were acquired on a LSR Fortessa flow cytometer (BD Biosciences, DIVA Version 8.x.).

### Detection of IFN-γ and granzyme B by FluoroSpot assay and ELISA

The functional activity of seATCs was determined after STE with the Asp-lysate using FluoroSpot assay and quantitative enzyme-linked immunosorbent assay (ELISA). The FluoroSpot assay was performed according to the manufacturer’s instructions (Mabtech, Nacka Strand, Sweden) to simultaneously detect the secretion of IFN-γ and granzyme B (GzB). Twelve days Asp-lysate-expanded seATCs were plated at a density of either 2.5 × 10^5^ or 5.0 × 10^5^ cells/well on pre-coated FluoroSpot plates (in RPMI1640/10% AB serum) and were incubated for 48 hours. The anti-CD28 mAb was included to enhance the stimulation. Negative and positive controls were carried out by using either solitary medium or anti-CD3 mAb according to the manufacturer’s instructions. Spots identified with the filter for FITC represented IFN-γ producing cells and spots identified by the filter for Cyanine 3 (Cy3) detected granzyme B producing cells. Results were expressed as spw, equal to the number of spots in the antigen well after subtracting those of the respective negative control well. Furthermore, the granzyme B ELISA was performed according to the manufacturer’s instructions (both eBioscience) in which secretion levels of both effector molecules were detected in the supernatants of expanded seATCs on day 12 of STE.

### Immunophenotyping of PBMCs and seATCs

The viability of PBMCs and seATCs (2.5 x 10^5^) was addressed by their appropriate position in the forward scatter (FSC) versus side scatter (SSC) plot as described previously (19). The cell number was determined *via* single platform (without washing steps) by using TruCount tubes (BD Biosciences) and cells were analyzed for the memory T-cell subsets. The cell number for T-helper (Th) cells was determined *via* a dual platform (total white cell count from a hematology analyzer (Sysmex, Kobe, Hyogo, Japan) and leukocyte subsets percentages by flow cytometry. T-cell subsets were defined as follows: naïve; CD62L^+^/CD45RA^+^, central memory (T_CM_); CD62L^+^/CD45RA-, effector memory (T_EM_); CD62L-/CD45RA-, and terminally differentiated effector memory expressing CD45RA^+^ (T_EMRA_); CD62L-/CD45RA^+^. The definition of Th1, Th2, T-follicular helper (TFH), Th9, Th17 and Th22 subsets [according to OMIP 042 (40)] is shown in the **Supplementary Figure 3**, respectively. The following antibodies were used to perform appropriate multi-parameter flow cytometric analysis of Th cells: CCR4 BV421, CD3 BV510, CXCR5 BV605, CD4 BV650, CD161 BV711, CD45RA BB515, CCR10, PerCP CY5.5, CCR6 PE, CXCR3 PE-CF594, CD62L Pe-Cy7, PD1-Alexa Fluor647, CD8 APC R700, CD45 APC H7 (all from BD Biosciences). Samples were acquired on a LSR Fortessa and a LSR II flow cytometer, and the FACSDiva software Version 8.x (all BD Biosciences) was used for analysis and data evaluation.

### Expansion of CSA-enriched Asp-specific T cells

Asp-specific T cells either against the Asp-lysate or PepMix enriched *via* CSA (csaATCs) were seeded into 96 well plates at 1 x 10^4^ cells per well in TexMACS (Miltenyi Biotec). For feeder cells, autologous PBMCs were irradiated at 50 Gy and seeded at 1 x 10^6^ cells per well. Cultures were supplemented with recombinant human Interleukin-2 (rhIL-2, 50 IU/ml; Miltenyi Biotec) and maintained for 12 days including regular media change and splitting of the cells.

### Antifungal activity of seATCs

*Aspergillus fumigatus* (ATCC 204305) was cultured in Malt Extract agar (MEA, Sigma) plates for three to four days at 37°C. Spores were collected in PBS with Tween 20 (0.1%) and the conidia suspension was filtrated using a 40 μm cell strainer (Falcon). The concentration was estimated on the CASY cell counter (Roche-Innovatis) and *A. fumigatus* conidia in a density of 1.2 x 10^4^cells/well were added to 96-well flat-bottom treated plates (Cytone One) in RPMI 1640 medium (Gibco), buffered with MOPs (Sigma) and supplemented with 2% glucose, followed by incubation of 12 hours to allow germination. 0.2 x 10^6^ Asp-lysate or PepMix-specific seATCs/well or either PepMix (positive control) or CMVpp65 peptide pool (negative control, Miltenyi Biotec) were incubated for six hours. Cell were washed in PBS to stop the inhibitory activity and non-adherent cells were removed, followed by the addition of XTT (2.3-bis(2-methoxy-4-nitro-5-sulfophenyl)-5-[carboyl(phenylamino)]-2H-tetrazoliumhydroxide) to determine fungal viability by assessing mitochondrial enzyme activity (41). The calculation used to assess viability was as follows: [(A450 of hyphae incubated with seATCs/PepMix/CMVpp65-A450 of seATCs/PepMix/CMVpp65) X100/A450 hyphae live]. For imaging, plates were prepared with 1.2x10^2^ fungal cells/well, following the same conditions mentioned above. The staining was carried out with calcofluor white (CFW) (24 µg/ml), and the images were retrieved using a Thermo Scientific Invitrogen EVOS FL microscope. The XTT reduction assay was performed as previously described (42). Briefly, 100 µl of XTT solution (1 mg/ml) added 8 µl of menadione solution (1 mM) were added to each well, followed by incubation at 37°C for two to three hours. After the incubation time, 80 µl supernatant was transferred to a flat-bottom 96-well plate, and the absorbance at 450nm was determined using the multimode plate reader (Victor Nivo).

### Cytotoxicity assay

Autologous PBMCs were labeled using the CellTrace Violet Cell Proliferation Kit (Invitrogen, Waltham, MS USA), seeded into 24 well plates and loaded with the appropriate antigen (Asp-lysate, single peptide pools or PepMix, as mentioned above). Unloaded PBMCs served as control for unspecific killing. Expanded csaATCs and seATCs were harvested, counted and seeded into 96 well plates together with unloaded or loaded PBMCs in different effector-to-target ratios (1:1, 5:1, 10:1). As a control for target cell viability, target cells were incubated in absence of T cells. Overnight co-culture supernatants were collected for subsequent multiplex analysis. Cells were washed and stained with 7-AAD and analyzed *via* flow cytometry using the BD FACSCanto 10c system.

### Cytokine/Chemokine measurement in cell culture supernatants

Cell culture supernatants collected from target-cell depended cytotoxicity assays were subjected to LEGENDPlex using the CD8/NK Cell Kit (BioLegend). Samples were prepared and acquired using the BD FACSCanto 10c system according to the manufacturer’s instruction.

### Statistical analysis

**Figure 3:**
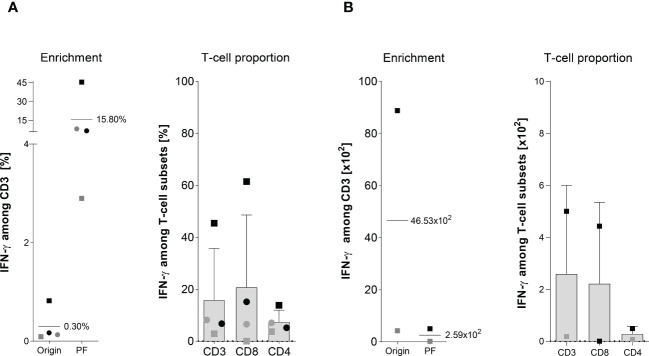
Magnetically enrichment of PepMix-stimulated ATCs. The enrichment efficiency of in vitro stimulated Asp-specific T cells was assessed by magnetic selection using the IFN-γ cytokine secretion assay (CSA). 1 x 10^7^ isolated PBMCs were stimulated overnight with the pooled overlapping peptide pools from Asp and *Candida albicans*-(PepMix: CatB, Crf1, f22, Gel1, pmp20, SHMT, SOD and MP65) each pool at a final consternation of 1 µg per peptide/ml while unstimulated PBMCs served as the negative control. Aliquots of the respective cell fractions collected before (original fraction, Origin) and after enrichment (positive fraction, PF) were used for detailed characterization of activated T-cell subsets by multicolor flow cytometry. **(A)** Frequency of IFN-γ^+^ Asp-specific CD3^+^ T cells before (Origin) and after magnetic selection (PF) and the proportion of IFN-γ^+^ Asp-specific T-cells among CD3, CD4 and CD8 T cells after magnetic enrichment in the PF are shown. Results are displayed as individual results (n=4) and mean ± SD. **(B)** Absolut cell numbers of IFN-γ^+^ Asp-specific CD3^+^ T-cells and among the different subsets (CD3, CD8 and CD4) are shown for two experiments, as insufficient cell counts after enrichment did not permit further analyses visualized by the respective mean ± SD. Squares and circles represent the same individuals of our healthy donor cohort (n=320).

Statistical analysis was performed using the Prism V7 and V8 software (GraphPad, San Diego, California, USA). The results are displayed as mean ± standard deviation (SD). Data were analysed using either T-Test (**Figure 2**) Mann-Whitney U-Test (for **Figures 1**, **3**) or Wilcoxon test for the remaining figures to determine the statistical significance between the different parameters. Significance levels were calculated and expressed as p-values (*p < 0.05, **p < 0.01, ***p < 0.001, ****p<0.0001).

## Results

### Screening of healthy donors from the alloCELL registry for the presence of Asp-specific T cells based on Asp-lysate and overlapping peptide pools

First, we screened 320 blood samples from healthy volunteer donors (HVDs), recruited from the alloCELL registry (43), for the presence of ATCs. Asp-specific T-cell responses were assessed in PBMCs stimulated with Asp-lysate *via* the IFN-γ EliSpot assay. IFN-γ-secreting ATCs were detectable in 65% of all HVDs with Asp-specific T-cell responses ranged from 2.0 to 238.5 spots per well (spw)/2.5 x 10^5^ PBMCs (mean 11 spw, detection limit: > 2 spw) (**Figure 1A**). Additionally, 30 out of 320 HVDs were tested for specific T-cell responses against the recently described single Asp-specific peptide pools (Cat, Crf1, f22, Gel1, pmp20, SHMT, and SOD) and the *Candida albicans*-specific peptide pool MP65 (**Figure 1B**). IFN-γ^+^ ATCs were observed in 86.7% of tested HVDs after Asp-lysate stimulation with a mean T-cell response of 14 spw and a range of 0.5 to 102.5 spw (**Figure 1A**). Stimulation with the single peptide pools resulted in the detection of specific T-cell responses in only 3.3 to 20% of the HVDs with mean T-cell responses ranging from 0.4 to 4.0 spw (**Figure 1B**). Nevertheless at least peptide-pools against SHMT and MP65 (Candida), showed high response rates observed in 20% (mean 1.5 spw) and 40% (mean 4.0 spw) of all 30 HVDs, respectively (**Figure 1B**). On the basis of IFN-γ secretion, a higher number of HVDs responded to the stimulation with Asp-lysate than to single peptide pools.

### Magnetic enrichment of ATC *via* small-scale CSA using the Asp-lysate

Next, we focused on the direct-magnetically enrichment of ATCs by IFN-γ CSA (csaATCs) representing the small-scale process largely analogous to the GMP-compliant clinical large-scale IFN-γ CCS manufacturing process. 1 x 10^7^ PBMCs from eight HVDs were stimulated for 16 hours with Asp-lysate. **Supplementary Figure 1A** shows the result of one representative experiment. Stimulation resulted in the mean frequency of 0.28% IFN-γ^+^ csaATCs among CD3^+^ T cells (range of 0.07 to 0.9%) in the original fraction (Origin, before enrichment) (**Figure 2A**). IFN-γ^+^/CD3^+^ T cells were detected with a mean frequency of 49.6% +/- 21.7% in the positive fraction (PF) after enrichment (**Figure 2A**). Overall, Asp-specific T cells with a starting frequency ≥ 0.07% of IFN-γ^+^/CD3^+^ T cells in the Origin were successfully enriched in a range between 17.8% and 84.5% for all donors tested. However, a higher starting frequency of IFN-γ^+^/CD3^+^ T cells was not directly correlated to a higher enrichment efficiency in the PF, in which a higher enrichment of csaATCs could also be achieved for some donors with a lower starting frequency. The mean percentages of IFN-γ^+^ csaATCs among CD8^+^ and CD4^+^ T cells within the PF were 40.3% +/- 6.5% and 51% +/- 5.8%, respectively (**Figure 2B**). In addition, we analyzed the absolute cell number of IFN-γ^+^ csaATC among CD3^+^, CD8^+^ and CD4^+^ T cells within the PF, resulting in a mean of 19 x 10^2^ (range: 0 to 9.1 x 10^3^), 6.8 x 10^2^ (range: 0 to 4.4 x 10^3^) and 7.0 x 10^2^ (range: 0 to 1.7 x 10^3^), respectively, representing a recovery of 20% (**Figures 2C, D**). Although the mean absolute cell number of CD8^+^ and CD4^+^ csaATCs were not significantly different (683 vs 502, respectively), we observed a slightly higher enrichment of CD4^+^ T cells in five out of eight (63%) samples. It should be mentioned here that for the production of clinically applicable T cells using the IFN-γ CCS and enrichment on the CliniMACS Prodigy device, the initial cell number is 100 times higher, which should also result in a 100-fold increased yield in the clinical product (44). Besides IFN-γ, ATCs have also been shown to express Tumor Necrosis Factor (TNF)-α, CD137 (TNFR family molecule 4-1BB) and CD154 (CD40L) after Asp-lysate stimulation (45). This expression pattern might be exploited to catch a broader spectrum of ATCs. Therefore, ATCs from additional three HVDs were magnetically enriched based on different activation markers (IFN-γ, TNF-α, CD137 and CD154). As shown in **Figures 2E–G**, IFN-γ^+^ (22.0% +/- 11.8), TNF-α^+^ (5.3% +/- 4.1%) and CD137^+^ (12.4% +/- 7.3%) secreting CD3^+^ ATCs were successfully enriched from all three HVDs, whereas enrichment through the activation marker CD154 was insufficient (0.7% +/- 0.5%). The highest frequencies of IFN-γ^+^ ATCs were observed within the CD4^+^ (26.4% +/- 15.5%) T-cell population, whereas CD137 was mainly expressed within the CD8^+^ (31.6% +/- 24.0%) T-cell population. Next, we tested the distribution of different memory subsets within the positively selected ATCs. CD3^+^ ATCs revealed a strong increase of effector memory (T_EM_) T cells, independent of the activation markers used (**Figure 2H**). In contrast, compared to the original fraction, the percentage of T_EM_ - known to be responsible for long-term immunity - were only increased within the CD137^+^/CD8^+^ T-cell population. In conclusion, cells generated after stimulation with the Asp-lysate showed mainly a T_EM_ and late differentiated effector memory CD45RA^+^ T-cell (T_EMRA_) phenotypes (**Figures 2I, J**).

### Magnetic enrichment of Asp-specific T cells against the PepMix *via* IFN-γ CSA indicates the need for suitable donor selection procedures

Because ATCs were detectable in low frequencies after stimulation with single peptide pools (**Figure 1B**) when compared to the Asp-lysate, we tested a mixture of the peptide pools composed of CatB, Crf1, Gel1, pmp20, SHMT, SOD, and MP65 (termed “PepMix”) as a stimulus for magnetic enrichment *via* IFN-γ CSA. **Supplementary Figure 1B** shows one representative example for direct-magnetically enrichment of ATCs stimulated with the PepMix *via* IFN-γ CSA. Stimulation of 1 x 10^7^ PBMCs from HVDs (n=4) with the PepMix for 16 hours resulting in the activation of IFN-γ^+^ csaATCs among CD3^+^ T cells in a mean frequency of 0.3% (range of 0.09 to 0.8%) in the Origin (**Figure 3A**). Compared to the Asp-lysate, stimulation with the PepMix leads to a lower enrichment efficiency with a mean frequency of 35.3% IFN-γ^+^ csaATCs among CD3^+^ T cells in the PF (range between 2.9 to 45.4%) in which only one out of four runs resulted in a enrichment of > 10% IFN-γ^+^ csaATCs among CD3^+^ T cells. After enrichment, the proportion of IFN-γ^+^ csaATCs in the PF was higher within CD8^+^ T cells (20.8% +/- 27.8%) than in CD4^+^ T cells (7.5% +/- 4.5%). Absolute cell number of IFN-γ^+^ csaATCs within the PF, resulting in a mean of 2.6 x 10^2^ (CD3^+^), 2.2 x 10^2^ (CD4^+^), and 0.3 x 10^2^ (CD4^+^), respectively, representing a recovery of 56% (**Figure 3B**). Overall, stimulation with the PepMix lead to a comparable frequency of IFN-γ^+^ csaATCs as stimulation with the Asp-lysate, but with a significantly lower enrichment efficiency for the majority of donors tested. Our IFN-γ-CSA data show that a high enrichment efficiency is not necessarily dependent on a specific ATC starting frequency. Therefore, investigation of enrichment efficiency in the pre-selection of T-cell donors in addition to the IFN-γ^+^ ATC starting frequency is crucial for the manufacturing of clinical-grade T-cell products in sufficient cell numbers and purity.

### Short-term *in vitro* expansion of Asp-specific T cells with Asp-lysate and peptide pools

**Figure 4:**
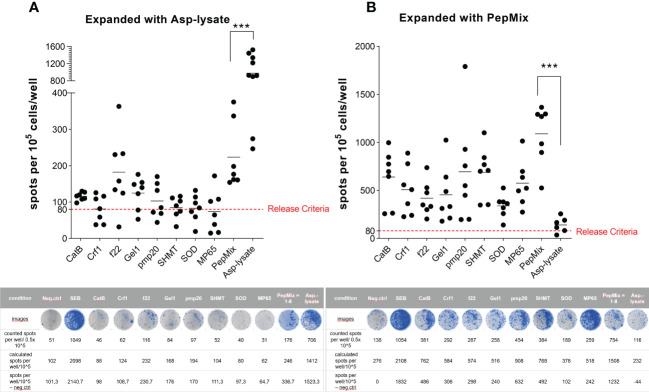
Functional activity of short-term expanded (STE) ATCs analyzed via IFN-γ EliSpot assay. PBMCs were short-term expanded (STE) for 12 days with either the **(A)** Asp-lysate (50 µg/ml) or the **(B)** combination of eight fungal peptide pools termed PepMix (1 µg per peptide/ml per peptide pool, composed of: CatB, Crf1, f22, Gel1, pmp20, SHMT, SOD and MP65 peptide pool). A) Numbers of IFN-γ spots per 10^5^ cells/well of lysate-expanded ATCs (including mean values) after restimulation for 16 hours with either Asp-lysate (n=9), single peptide pools (n=7), or PepMix (n=7), as indicated, are shown. B) Numbers of IFN-γ spots per 10^5^ cells/well of PepMix-expanded ATCs (including mean values) after restimulation for 16 hours with either the Asp-lysate (n=6), single peptide pools (n=7), or the PepMix (n=7), as indicated, are shown. For analyses of spots per well, background values were subtracted. The cut-off criterion (red dotted line) for a clinical product were set to 80 spots/10^5^ cells. Data are shown in total as individual result and mean of healthy individuals from our cohort of donors (n=320). Representative EliSpot results are shown below the respective graph. Statistically significant difference is indicated by (***p < 0.001).

The magnetic enrichment of ATCs *via* the IFN-γ CSA was in general successful if the Asp-lysate or the PepMix was used as stimuli. Starting with a comparable mean frequency of IFN-γ^+^ csaATCs among CD3^+^ T cells in the Origin (mean 0.3% for both stimuli before enrichment) resulted in a higher enrichment efficiency using the Asp-lysate (**Figure 2A**) when compared to the PepMix (**Figure 3A**). Since csaATCs were enriched only at low frequencies and total cell numbers after stimulation with the PepMix using the IFN-γ CSA as direct isolation procedure in most donors tested, it was examined whether the short-term expansion (STE) method (15, 19) could increase the yields of ATCs. PBMCs of HVDs were initially pulsed for 12 days with either the Asp-lysate (n=9), or combined peptide pools (PepMix; n=7) in combination with IL-15, added at day nine. After the expansion period, cell cultures were restimulated either with the single peptide pools, the PepMix or the Asp-lysate, respectively to analyze their specificity *via* the IFN-γ EliSpot assay (**Figures 4A, B**). For Asp-lysate- and PepMix short-term-expanded ATCs (seATCs), a number of > 80 spots/10^5^ cells were classified as release criterion for positive responses based on similar release criteria shown to be safe in other studies (15, 20).

Cell cultures expanded and shortly restimulated (16 hours) with Asp-lysate (**Figure 4A**) showed the highest number of IFN-γ secreting seATCs (mean: 863 spw ± 154 spw, range: 205 to 1422 spots/10^5^ cells) and all HVDs fulfilled our release criterion (T-cell response > 80 spots/10^5^ cells). In contrast, if Asp-lysate expanded seATCs were restimulated with the individual peptide pools alone or with the PepMix, only restimulation with the peptide pool f22 (mean: 98 spw ± 47 spw, range: 0 to 283 spots/10^5^ cells) and the PepMix (mean: 139 spw ± 47 spw, range: 0 to 295 spots/10^5^ cells) resulted in responses above the release criterion in five out of seven (71%) and six out of seven HVDs (86%), respectively. Only few or even no Asp-lysate expanded seATCs showed T-cell responses above the release criterion after restimulation with the other investigated peptide pools (CatB, Crf1, Gel1, pmp20, SHMT, SOD, MP65) (**Figure 4A**). For PepMix-expanded seATCs (**Figure 4B**), highest number of IFN-γ secreting cells were detected after short restimulation (16 hours) with the PepMix (mean: 833 spw ± 138 spw, range: 332 to 1220 spots/10^5^ cells), in which all tested HVDs fulfilled the release criterion (T-cell response > 80 spots/10^5^ cells). The magnitude of the T-cell response triggered by PepMix expansion and restimulation was comparable to that induced by Asp-lysate expansion and restimulation. Also, restimulation with individual peptide pools resulted in high numbers of PepMix-expanded seATCs in most HVDs tested (**Figure 4B**) when compared to Asp-lysate-expanded seATCs. Interestingly, if Asp-lysate was used for the restimulation (n=6) of PepMix-expanded seATCs, none of those HVDs reacted to the Asp-lysate (**Figure 4B**).

To get more detailed information on the activation status of Asp-lysate-expanded or PepMix-expanded seATCs, respectively, we additionally examined the secretion of IFN-γ and TNF-α on CD3^+^ T cells using the intracellular cytokine staining (ICS) assay. CD4^+^ and CD8^+^ T-cell subsets were further evaluated and are shown in **Supplementary Figure 2**.

**Figure 5:**
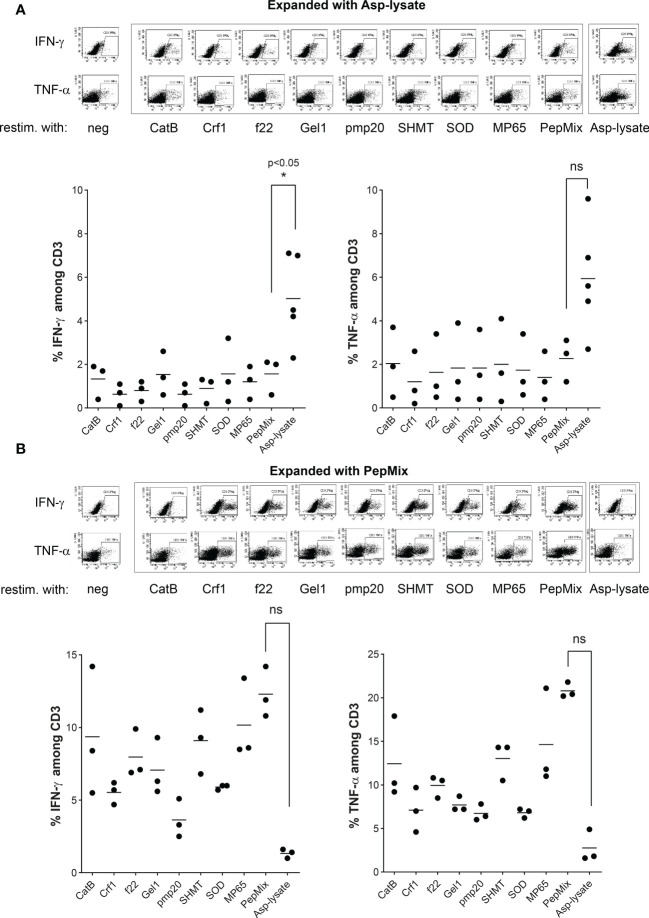
Intracellular cytokine staining of IFN-γ and TNF-α producing ATCs. PBMCs were short-term expanded (STE) for 12 days with either the **(A)** Asp-lysate (50 µg/ml) or a **(B)** combination of eight fungal peptide pools termed PepMix (1 µg per peptide/ml per peptide pool, composed of: CatB, Crf1, f22, Gel1, pmp20, SHMT, SOD and MP65 peptide pool) . Representative dot plots (gated on CD3^+^ T cells) and percentage values of IFN-γ^-^ and TNF-α^-^ expressing CD3^+^ ATCs, expanded with either (A) Asp-lysate or (B) PepMix and restimulated with indicated antigens (x-axis) are shown. Diagrams include the mean values obtained from n=3-5 individual HDVs from the cohort of donors (n=320), pre-tested for the presence of Asp-specific T cells. Dot plots highlighted in grey represent cells expanded and restimulated including the same antigen. Background values, based on unstimulated cells (neg) were subtracted. Data are shown in total as individual result and mean. Statistically significant difference is indicated by (*p < 0.05), ns – not significant.

In **Figure 5A**, Asp-lysate-based STE resulted in high percentages of IFN-γ- and TNF-α-secreting seATCs among CD3^+^ T cells after restimulation with Asp-lysate (IFN-γ: 5.0% ± 0.9%, TNF-α: 5.9% ± 1.2%). In contrast, the percentages of IFN-γ- and TNF-α-producing seATCs among CD3^+^ T cells was significantly lower if individual peptide pools or the PepMix, was used for restimulation. Vice versa, as shown in **Figure 5B**, PepMix-based STE resulted in higher percentages of IFN-γ^+^ and TNF-α^+^ seATCs among CD3^+^ T cells after restimulation with PepMix (IFN-γ: 12.0% ± 1.0%, TNF-α: 21.0% ± 0.5%) when compared to restimulation with Asp-lysate (IFN-γ: 1.3% ± 0.19%, TNF-α: 2.8% ± 1%). As expected, the percentage of IFN-γ^-^ and TNF-α-producing seATCs was also high after restimulation with individual peptide pools. Furthermore, PepMix-based STE resulted in a higher frequencies of IFN-γ-secreting CD3^+^ T cells when compared to Asp-lysate expanded seATCs. Comparing CD4^+^ and CD8^+^ T cells, frequency of TNF-α^+^ was higher in CD4^+^ T cells after expansion with the Asp-lysate, while IFN-γ was higher in CD8^+^ seATCs (**Supplementary Figure 2A**). Higher proportion of TNF-α and IFN-γ was observed for PepMix-expanded CD8^+^ T cells as compared to CD4^+^ T cells (**Supplementary Figure 2B**). In addition, restimulation with the same stimulus as for STE (Asp lysate or PepMix) resulted in the higher TNF-α and IFN-γ response. Generally, ICS data confirm the results obtained by IFN-γ EliSpot assay (**Figure 4**) for the expanded seATCs after short antigen restimulation.

According to our results it can be assumed that corresponding epitopes from the individual peptide pools are either underrepresented or even absent in the Asp-lysate and, conversely, that the Asp-lysate also contains epitopes that are not present in the individual peptide pools. Nevertheless, both types of stimuli (Asp-lysate and PepMix) could be used for the successful short-term expansion of seATCs of the respective cells are present in the starting material.

### Cytotoxic capacity of Asp-specific csaATCs and seATCs

**Figure 6:**
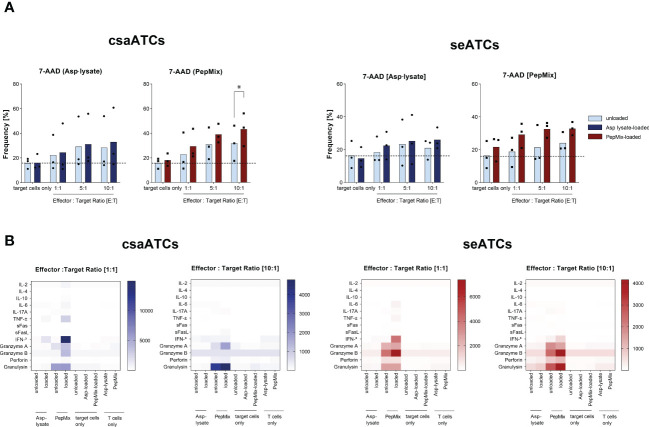
Cytotoxic potential of csaATCs and seATCs. Asp-specific T cells obtained by IFN-γ Cytokine Secretion Assay (csaATCs) and after short-term expansion (STE) for 12 days (seATCs) were investigated for their cytotoxic functionality. To generate csaATCs and seATCs, PBMCs were stimulated with either the Asp lysate (50 µg/ml) or a combination of eight fungal peptide pools called PepMix (1 µg per peptide/ml per peptide pool consisting of: CatB, Crf1, f22, Gel1, pmp20, SHMT, SOD and MP65 peptide pools). To obtain a sufficient number of csaATCs for the analyses, cells were additionally expanded for 12 days. With expanded ATCs, cytotoxicity tests were performed with unloaded and Asp-lysate- or PepMix-loaded autologous PBMCs as target cells. **(A)** Frequencies of cells with 7-AAD^+^ cells among target cells (CellTrace Proliferation dye positive). Light bars: unloaded target cells. Dark bars: loaded target cells. Results are displayed as individual results and as means (n=3). Statistical significance was calculated using Two-way ANOVA and multiple comparisons test (*p < 0.05). **(B)** Cell culture supernatants from cytotoxicity assays from day 12 for effector to target ratios of 1:1 and 10:1were analyzed with respect to presence of cytotoxic effector molecules by LEGENDPlex Assay. Results are displayed as heatmap. The results are expressed as the mean of the duplicates for samples of three tested donors, pre-tested for the presence of Asp-specific T cells (n=3/320). The values are represented by different colors, as referenced in the bar. As control, unloaded, Asp-lysate or PepMix-loaded target cells only as well as Aps-lysate-specific and PepMix-specific T cells only were used.

Asp-specific T cells enriched by IFN-γ CSA were expanded for 12 days using irradiated autologous PBMCs and were shown to recognize and kill target cells presenting antigens from the Asp-lysate or PepMix. Therefore, co-cultures of target cells loaded or unloaded with Asp-lysate or PepMix and the expanded csaATCs and seATcs were established. After overnight co-cultivation, the frequency of dead cells (7-AAD^+^) was determined using flow cytometry (**Figure 6A**). Specific killing was detected for both csaATCs as well as seATCs. Antigen-loaded target cells exhibited higher frequencies of dead cells (7-AAD^+^) compared to unloaded target cells. Regardless of the generation strategy, both csaATCs (E:T ratio 10:1, unloaed/loaded: 31.8%/43.4% 7AAD^+^ cells, *p<0.05) and seATCs (E:T ratio 10:1, unloaed/loaded: 24.1%/32.9% 7AAD^+^ cells) against the PepMix showed a higher killing efficiency compared to csaATCs (E:T ratio 10:1, unloaed/loaded: 28.4%/33.0% 7AAD^+^ cells) and seATCs (E:T ratio 10:1, unloaed/loaded: 21.1%/26.1% 7AAD^+^ cells) against the Asp-lysate. To investigate the ability of csaATCs and seATCs to produce and secrete cytotoxic mediators upon target recognition, cell culture supernatants of co-cultures were analyzed by LEGENDPlex (**Figure 6B**). Increased levels of effector molecules like TNF-α, IFN-γ, granzyme A, granzyme B, perforin, and ganulysin were detected in the supernatants of csaATC and seATC co-cultures with loaded target cells compared to unloaded target cells, indicating the cytotoxic potential of the cells. Comparable to flow cytometry data, PepMix-specific ATCs showed higher cytotoxic potential than Asp-lysate-specific ATCs. Higher concentrations of the investigated effector molecules were detected in the co-cultures with PepMix-specific ATCs compared to Asp lysate-specific ATCs and independent of the generation strategy (CSA or STE).

**Figure 7:**
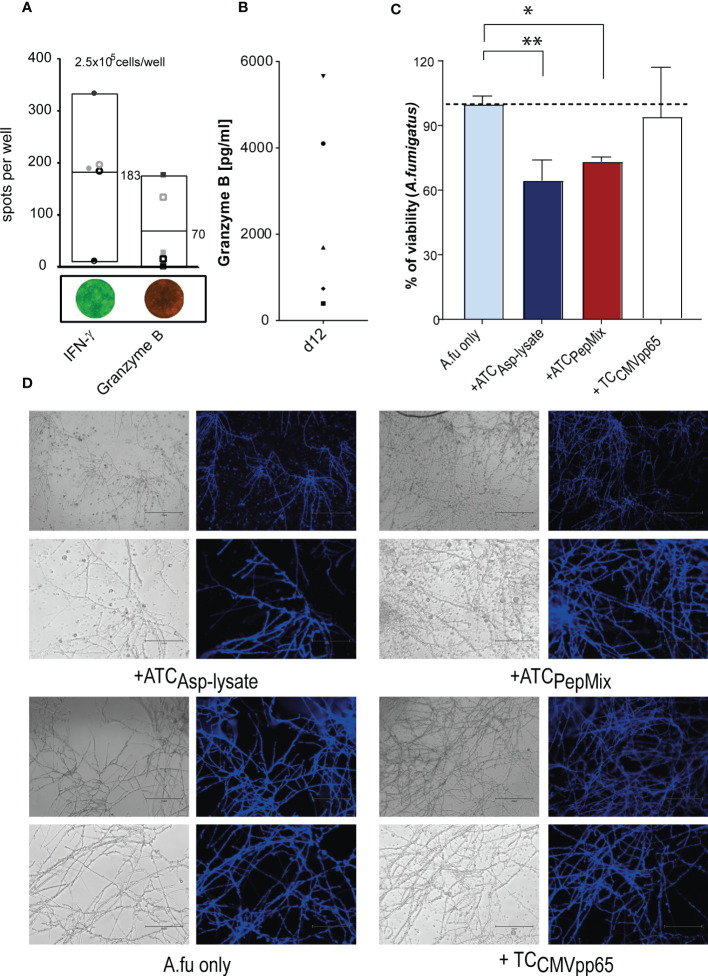
Functional and cytotoxic analyses of seATCs *via* FluroSpot assay and ELISA. PBMCs were short-term expanded (STE) for 12 days with Asp-lysate following Asp-restimulation for 16 hours with Asp-lysate (n=5). The cytotoxic activity of Asp-specific T cells was investigated by means of IFN-γ and granzyme B. **(A)** Numbers of IFN-γ and granzyme B positive spots per 2.5 x 10^5^ cells/well of ATCs with mean values represented *via* Box blots of five individual HDVs including representative images of IFN-γ (green) and granzyme B (red) secreting ATCs are shown. Results are given as the number of spots per well (spw), representing the number of spots in the antigen well after subtracting those of the respective negative control well. **(B)** The diagram shows individual values of granzyme B secretion (in pg/ml) in the supernatant of 12 days expanded ATCs, detected by ELISA (n=5). The different symbols represent the individuals ATCs from healthy donors who were pre-tested for the presence of Asp-specific T cells. **(C, D)**. *Aspergillus fumigatus* hyphae were either left untreated (Afu only) or incubated for six hours with CMVpp65 peptide pool expanded T cells (+TC_CMVpp65_), as additional control, or Asp-lysate (+ATC_Asp-lysate_) and PepMix-(+ATC_PepMix_) specific seATCs. Viability was analyzed by XTT reduction assay. The healthy individuals used belonged to our cohort of donors (n=320) who were pre-tested for the presence of Asp-specific T cells. Results are displayed as mean ± SD for samples from one representative healthy donor. Statistically significant difference is indicated by (*p < 0.05 and **p<0.01). A.fu, *Aspergillus fumigatus*; TC, T cells.

Furthermore, in order to test the cytotoxic potential of expanded seATCs, the secretion of effector molecules such as IFN-γ and granzyme B (GzB), of post-thawed Asp-lysate-expanded seATCs from five HVDs were analyzed *via* FluoroSpot assay. A T-cell release of IFN-γ (mean: 183 spw, range: 10 to 334 spots/10^5^ cells) was detected for all five seATCs while GzB (mean: 70 spw, range: 0 to 177 spots/10^5^ cells) was detected in four out of five ATCs (**Figure 7A**). In the FluoroSpot assay, the spots for GzB are significantly smaller compared to the IFN-γ^+^ spots. The analysis of seATC-responses after restimulation was significantly more difficult for GzB than for IFN-γ due to the small spot size and high background response of the negative control (seATCs without restimulation). This could be a possible reason why, after subtraction of the negative control, a specific T-cell response for GrzB was detected in only four of the five seATCs examined by FluoroSpot assay. However, analysis *via* ELISA showed high concentrations of secreted GzB levels for all five seATCs (mean: 2518 pg/ml; range: 395 to 5661 pg/ml) (**Figure 7B**), which suggests the cytotoxic functionality of Asp-lysate expanded seATCs from all tested donors.

### Antifungal activity of seATC against *Aspergillus fumigatus*

To determine the hyphae damage effect caused by seATCs generated by PepMix-based or Asp-lysate-based STE in a co-cultured germinated *A. fumigatus* conidia, we used the XTT reduction assay, as previously described (34, 42, 46). Treatment with Asp-lysate-specific seATCs resulted in a significant decrease in fungal viability about 35 ± 7.76% (**p<0.01, **Figures 7C, D**) in a similar way as observed for treatment with PepMix-specific seATCs, (26.74 ± 1.78, *p <0.05, **Figures 7C, D**). Moreover, results obtained after the seATC treatment displayed an evident impairment in hyphae ramification and branching. Although to a lesser extent, a compromise in hyphae development and apical growth was analyzed by the treatment with PepMix-specific seATCs. By this observation, it is conceivable that the hyphal damage promoted between each seATC treatment occurs in different responsive ways.

### Deep phenotyping of Asp-lysate- and PepMix-specific seATCs

**Figure 8:**
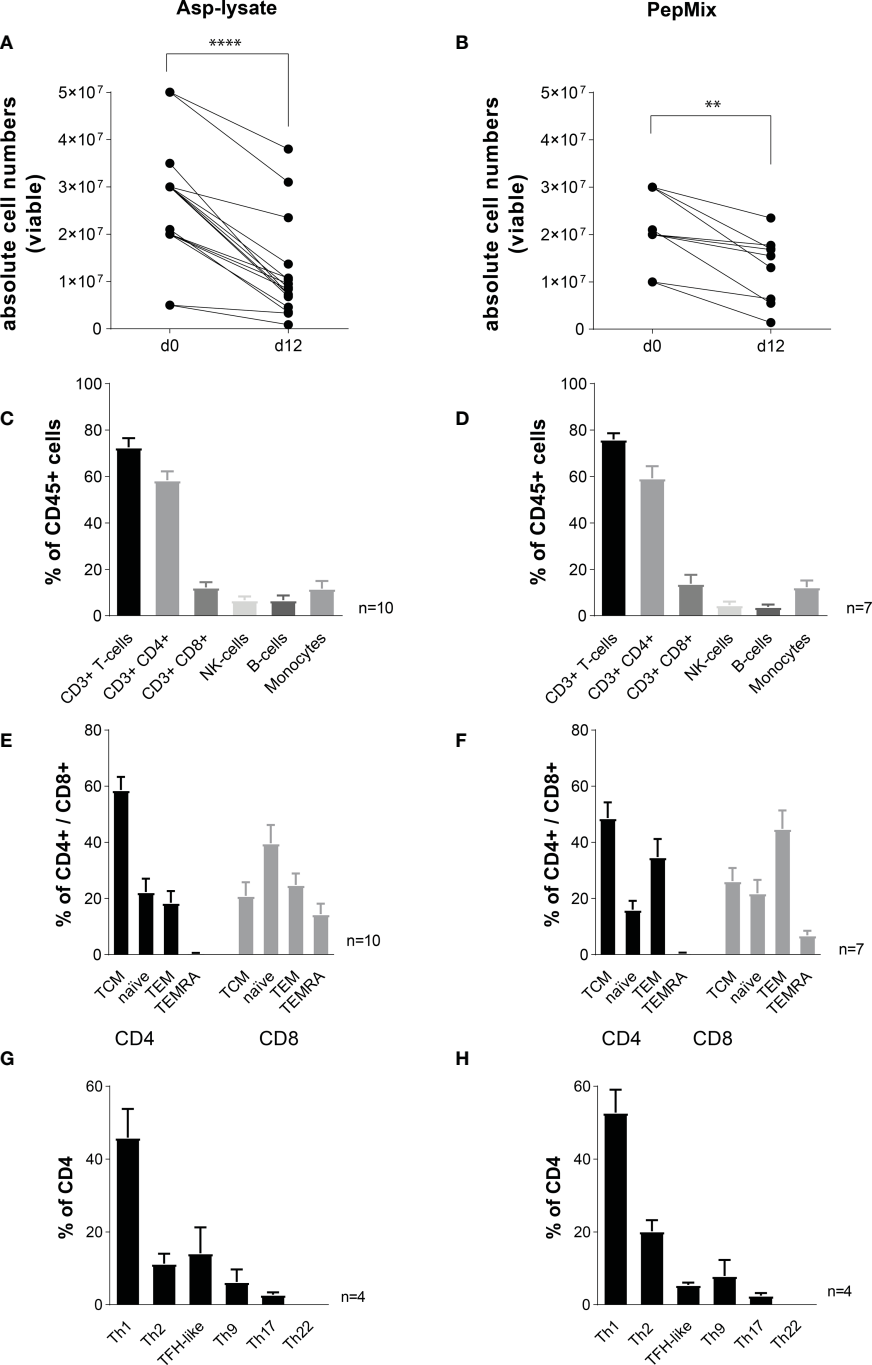
Immunophenotyping of short-term expanded seATCs. PBMCs were short-term expanded (STE) for 12 days with either the **(A, C, E, G)** Asp-lysate (50 µg/ml, n=10) or the **(B, D, F, H)** PepMix (1 µg per peptide/ml per peptide pool, composed of: CatB, Crf1, f22, Gel1, pmp20, SHMT, SOD and MP65 peptide pool, n=7). Absolute cell numbers per well before (day 0) and after expansion (day 12) for seATCs expanded with **(A)** Asp-lysate- or **(B)** PepMix are shown. Statistically significant difference is indicated by (**p < 0.01 and ****p<0.0001). **(C, D)** Mean proportion of CD3^+^, CD4^+^ and CD8^+^ seATCs, NK cells, B-cells, and monocytes within CD45^+^ cells after expansion with **(C)** Asp-lysate or **(D)** PepMix are shown as mean ± SD. **(E, F)** Immune phenotypes of CD8^+^ and CD4^+^ seATCs and **(G, H)** CD4^+^ T-cell subsets, expanded with Apsp-lysate or PepMix are shown as mean ± SD. The healthy individuals used belonged to our cohort of donors (n=320) who were pre-tested for the presence of Asp-specific T cells.

Although seATCs were highly expanded within 12 days (**Figures 4A, B**), the absolute number of PBMCs within the G-REX device was in mean 3.2-fold decreased after STE with the Asp-lysate (****p<0.0001) and 2.5-fold after STE using the PepMix (**p<0.01) (**Figures 8A, B**). Nevertheless, for clinical use, the number of starting PBMCs can be easily adapted to 1 x 10^8^ or to achieve at least 3 to 4 x 10^7^, which was recently shown to be a sufficient cell number for the treatment of adult patients (23). We did not detect any significant difference in viability between the two expansion methods as shown in **Supplementary Figure 3**. Both STE with Asp-lysate and PepMix resulted in a similar percentage of CD3^+^ T cells (72.6% vs. 76%), CD3^+^/CD4^+^ T cells (58% vs. 59%), CD3^+^/CD8^+^ T cells (12.3% *vs.* 13.8%) and a similar distribution of NK cells (6.7% *vs.* 4.6%), B cells (6.7% vs. 3.8%) and monocytes (11.7% vs 12.4%) (**Figures 8C, D**). Following STE with Asp-lysate or PepMix, CD4^+^ T cells were mainly of T_CM_ phenotype (58.7% *vs.* 48.7%), but included also T cells of naïve (T_N_) (22.3% vs. 16%) and T_EM_ phenotypes (18.5% vs. 34.8%) with nearly no T_EMRA_ (0.5% *vs*. 0.5%) (**Figures 8E, F**). Although the CD8^+^ T cells mainly comprised T_N_ (40%) after Asp-lysate and mainly T_EM_ (44.9%) after PepMix STE, CD8^+^ T cells remained a minor population after the expansion procedure. Both stimuli generated high percentages of early and late-differentiated memory T cells within the CD4^+^ and CD8^+^ T-cell population.

Despite the impact of CD8^+^ T cells, especially the importance of different CD4^+^ Th subsets in controlling IFDs has been shown previously (47). Therefore, we extended our flow cytometry panel to enable a basic classification of CD4^+^ Th cells of seATCs in more detail, based on the following subsets: Th1, Th2, Th17, Th9, and Th22, and follicular helper T cells (TFH-) (see **Supplementary Figure 1** for gating strategy). As expected, Th1 cells were predominantly present in both Asp-lysate (mean: 20.3%) and PepMix (mean 24.5%) seATCs. In contrast, Th2 (5.6% vs. 6.6%), TFH (vs 5.4% vs 14.1%), Th17 (2.1% *vs.* 2.6%) and Th9 (5.4% vs. 7.5%) T-cell subsets represented minor populations within Asp-lysate and PepMix seATCs (**Figures 8G, H**). Th22 subsets could not be detected (**Figures 8G, H**). Thus, seATCs consisted predominantly of Th1 polarized CD4^+^ T cells with a memory phenotype.

In summary, our results indicate that the generation of ATCs for the treatment of IFD could be achieved by immediate treatment using IFN-γ CCS-derived ATCs (ccsATCs) and sustained treatment using STE-derived seATCs from the same starting material. As both methods were performed with the same starting material, we demonstrated that functional ATCs could be enriched and generated using both techniques. Overall, both methods showed better reactivity and yield with the Asp-lysate. The results of our study also show that pre-testing of potential donors using the later method and antigen to produce clinically applicable T cells is essential to generate products in high quantity and quality.

## Discussion

Immunocompromised patients after HSCT are at high risk for, invasive fungal infections caused by *Aspergillus ssp*. and *Candida ssp* (48). Current antifungal prophylaxis is more potent to prevent Candida infections than Aspergillus infections and the guidelines for the treatment of invasive candidiasis, aspergillosis and mucormycosis in leukemia and transplant patients (ECIL-6 guidelines) provide the basis for the first line treatment of IA, exclusively based on antifungal agents (49). However, antifungal agents show limited clinical efficacy, are cost intensive, and bear the risk of drug interactions and toxic side effects (7, 50). Therefore, the treatment of IFDs remains a clinically challenging problem and raises the questions of combining antifungal drugs with T-cell based immunotherapy.

The rationale for the adoptive transfer of ATCs was based on a single clinical trial in which Asp infection was successfully controlled in patients after haploidentical HSCT (22). In this study, more than 1200 Aspergillus-specific CD4^+^ T-cell clones were established from ten donors. Infusion of Asp-specific type-1 CD4^+^ T-cell clones resulted in the control of Aspergillus antigenemia and helped to clear invasive aspergillosis in a mean of 7.8 ± 3.4 weeks in nine of ten patients. None of the patients developed acute or chronic GvHD, or showed any other infusion-related toxicity after adoptive Asp-specific T-cell transfer in doses escalating from 1 × 10^5^ to 1 × 10^6^ cells/kg. This encouraging trial prompted several groups to develop successful but rather laborious protocols to generate ATCs by either direct immunomagnetic selection alone based on the expression of CD137 (45), or in combination with a subsequent *in vitro* expansion period to further enrich IFN-γ^+^ (24, 26, 46) or CD154^+^/CD137^+^ (30) ATCs. All of them used mainly antigen presenting cells (APCs) as feeders for their laborious expansion procedures of 2-4 weeks. Others focused on the *ex vivo* expansion alone without immunomagnetic pre-selection, but used dendritic cells (DCs) as feeders and either lysates (25), or only a single antigen based on the f16 peptide pool (28), Crf1 (28) or a combination of three peptide pools Crf1, Gel1 and Pmp20 (30). One reason for these lengthy and complicated protocols is the very low or even undetectable precursor frequency of ATCs in peripheral blood, similar or even rarer than those of AdV-specific T-cells (19). So far, these data have been mainly based on few donors tested for the presence of ATCs (34, 51). To get a more robust estimate of the precursor frequency against Asp in the healthy population, we screened 320 HVDs. Of these, 65% were positive for IFN-γ secreting ATCs, if PBMCs were stimulated with the Asp-lysate. This is in contrast to others showing no or even a lower percentages of donors with detectable ATCs (34, 51). In part, this discrepancy could be explained by the low number of HVDs tested in these studies. Nevertheless, although the percentage of HVDs positive for ATCs was quite high in our cohort, the frequencies of ATCs among PBMCs spread over a wide range (2 to 70 spw/10^5^ cells), indicating a high diversity of Asp-specific T-cell responses between individual healthy donors.

Since also peptide pools had been used successfully as stimuli to generate ATCs (30, 34, 51), we additionally screened 30 HVDs for specific T-cell responses against the Asp-lysate and, for the first time, eight individual Asp-specific peptide pools, respectively. Indeed, 86.7% of the 30 HVDs showed reactivity against the Asp-lysate after brief overnight stimulation, and only up to 20% of the HVDs were positive after stimulation with single peptide pools. Among these, only the SHMT peptide pool elicited detectable ATCs responses in 11 out of 30 HVDs while only a low number of responders had specific T-cell responses against the other six investigated Asp-specific peptide pools. In line with our results, others have also shown that the SHMT pool might be among the most immunogenic Asp-specific peptide pools (34, 51). Recently, to overcome the low precursor frequencies and to avoid long-term cell culture, Papadopoulou et al. adapted their short-term expansion protocol, which was originally performed to expand multivirus-specific T cells to enable the generation of ATCs within 9-11 days (34). A comparable method for the production of VSTs, but with a different stimulation procedure, was developed in our laboratory and applied as a first in man treatment under “named-patients-use” conditions (19). Nevertheless, while some patients can be treated in time with seATCs, others are in urgent need of immediate adoptive transfer after the diagnosis of Asp infection (36). Since the number of ATCs that could be obtained by the rapid IFN-γ CCS process might not be sufficient for multiple administrations and, on the other hand, the STE with a duration of 2-3 weeks until product release is too lengthy, both methods were combined in this study. From some HVDs, PBMCs were separated into two parts of which one was used for the IFN-γ CSA, which represents the small-scale version of the clinical-scale IFN-γ CCS, and the other for the STE. This combined strategy will in future allow the production of two separate cell products from the same starting material at the same time – by GMP-compliant IFN-γ CCS process and STE, thereby avoiding the need for potential secondary donor request. In two previous studies, cells were first stimulated with a non-GMP-grade cellular product, selected by the IFN-γ CSA and then expanded for two weeks to obtain sufficient cell numbers of ATCs (24, 26). In contrast, we used the recently purchasable GMP-conform Asp-lysate and for the first time, tested the stimulatory capacity of eight different fungi-specific peptide pools in combination in the IFN-γ CSA. Others had tested three peptide pools for the selection of ATCs, but only in combination with the enrichment *via* the activation markers CD137 and CD154 (30, 51). In our study, the mean purity and cell number of Asp-lysate-stimulated IFN-γ^+^/CD3^+^ csaATCs reached 49% and 19 x 10^2^ cells after magnetic enrichment, respectively, which were similar to or even higher than the values observed by Tramsen et al. (26) However, the enrichment of IFN-γ^+^/CD3^+^ csaATCs after stimulation with the PepMix was in 75% of the tested HDVs below 10%, suggesting the need of a suitable donor pre-selection to obtain sufficient quantities of ATCs for clinical application, especially for ATCs for which a large number of individuals showing low enrichment efficiency. Comparable to previous studies, we showed significant donor-to-donor variation in Asp-specific T-cell responses, with strong differences in the specific T-cell frequencies against antigens derived from the Asp-lysate and the investigated peptide pools. The difference between Asp-lysate and peptide pool stimulation is also reflected by the screening results of 30 HDVs, indicating that the corresponding epitopes from the individual peptide pools might be either underrepresented or even absent in the Asp-lysate and which may also be the case conversely. Furthermore it was shown that Asp-specific T-cell responses are directed against a multitude of different proteins, primarily against metabolically active *Aspergillus fumigatus* morphotypes. Antigens derived from membrane proteins were found to induce stronger Asp-specific T-cell responses when compared to antigens derived from cell wall or cytosolic proteins (52). For the Asp-derived proteins Crf1 and CatB, five and 30 different T-cell epitopes were identified previously, presented by different HLA-class II molecules (33). Our results, together with these previous studies, suggest that the Asp-specific T-cell response is largely heterogeneous. However, in this study we developed an effective strategy to enrich and generate Asp-specific T cells to improve adoptive T-cell therapy in immunocompromised patients with severe aspergillus-related complications. In this setting, similar to the use of the overlapping peptide pools, the use of the Asp-lysate allows stimulation of a wide range of specific T cells independent of single HLA alleles. The enrichment efficiency between T cells stimulated with Asp-lysate or peptide pools is not different when CD137 or CD154 instead of IFN-γ is used as shown by Stühler et al. (30) In our study we obtained the highest enrichment efficiency of Asp-specific CD3^+^ T cells by using IFN-γ followed by CD137 and TNF-α. CD154 resulted in the lowest enrichment efficiency. Nevertheless, it is important to mention that in the study of Stühler et al. it was demonstrated that the enrichment of CD154^+^ ATCs (median values of 2.9% among lymphocytes) was very low and that CD137^+^ ATCs (among CD4^+^ and CD8^+^ T cells) were contaminated with relatively high numbers of unspecific cells. A rather low enrichment efficacy of CD154^+^ ATCs was confirmed by our results. Although protection against Asp is mainly provided *via* CD4^+^ Th cells (12) with CD8^+^ T cells might also contribute to antifungal activity (53). Indeed, although some of the isolated IFN-γ^+^ csaATCs were CD8^+^, we observed a tendency towards CD4^+^ T-cell enrichment after Asp-lysate stimulation while PepMix-stimulation resulted in IFN-γ^+^ csaATCs with a tendency towards CD8^+^ T cells. Detailed analyses revealed that both IFN-γ^+^/CD4^+^ as well as IFN-γ^+^/CD8^+^ csaATCs enriched by IFN-γ CSA consisted mainly of effector memory and low percentages of central memory or naïve T-cell populations. In addition to purity, it is primarily the total cell number of enriched CD3^+^ T cells that determines the clinical applicability of the selection method used. After the IFN-γ CSA, we were able to enrich in mean 19 x 10^2^ IFN-γ^+^/CD3^+^ ATCs after Asp-lysate stimulation. An upper safety limit of total 25,000 CD3^+^ cells/kg body weight of allogeneic T cells has been established in the mismatch setting in order to avoid induction of GvHD (reviewed in Käuferle et al. (16)) and recently, even a minimum of approximately 150 IFN-γ^+^/CD3^+^ VSTs/kg was shown to be sufficient for successful adoptive transfer (16). Whether this might also be suitable for ATCs for treatment remains to be elucidated. At least the dose between 10^5^ to 10^6^ ATCs/kg showed clinical control of Aspergillus antigenemia and infectious mortality in the only clinical trial performed so far (22). It is also important to mention that these adoptively transferred ATCs showed expansion *in vivo* thereby assuming that even lower initial cell numbers might be sufficient. Under potential large-scale conditions using the IFN-γ CCS and the CliniMACS Prodigy (starting with 1 x 10^9^ leukocytes), it might be possible to enrich up to 19.2 x 10^4^ IFN-γ^+^/CD3^+^ ATCs. This is equivalent to 3800 IFN-γ^+^/CD3^+^ ATCs/kg body weight, if calculated with a patient body weight of 50 kg. Even if the cell numbers that can be obtained in this way are not sufficient to permanently control infection *in vivo* and overcome IFD, this could be a valuable option to bridge the time until the cells generated simultaneously in the STE are then ready for use at a later time point. This would be easily possible, because normally the results from the IFN-γ CCS process are already available within 24 hours after the end of leukapheresis and then the STE could still be started with cells from the same starting material.

To check this possibility, PBMCs of same HVDs were used to generate short-term expanded seATCs. We adapted our recently published protocol enabling the generation of fast, simplified and GMP-compliant VSTs within 9-12 days by using overlapping peptide pools in combination with IL-15 (15). Another successful method used by others is based on IL-4 and IL-7 to generate multivirus-specific T-cells (54). Both STE methods were successfully applied on patients to treat AdV (19, 32) or multiple viruses (20).

More recently, Papadopoulou et al. (34) adapted their multivirus-specific expansion protocol to produce ATCs by stimulating PBMCs with either a GMP-grade Asp-lysate or a mix of three different peptide pools covering the sequence of Crf1, Gel1 and SHMT. Independent of the stimuli used, functional active ATCs could be produced within 9-11 days and their safety was very elegantly assessed within a xenograft mouse model (34). Although our STE protocol shares certain features with the protocol by Papadopoulou et al. (34), the stimulation procedure itself by combining eight instead of three peptide pools and the addition of delayed IL-15, differs considerably. As expected, re-exposure of cells expanded ofer 12 days, with the respective initial stimuli (Asp-lysate or PepMix) resulted in high T-cell responses as measured *via* the IFN-γ EliSpot assay. In contrast, PepMix-expanded seATCs, re-stimulated with Asp-lysate completely failed to respond. On the other hand, seven out of eight Asp-lysate-restimulated ATCs reacted against the PepMix-expanded seATCs, albeit less extensive compared to those expanded with the Asp-lysate. As already mentioned, the underrepresentation of individual epitopes or even their absence in the corresponding stimulus could be the reason for the poor or missing responses after seATC restimulation. Generally, these data were also confirmed by ICS assay with the exception, that Asp-lysate-expanded ATCs did not show any response after PepMix restimulation which could be at least partially explained by the rather low number of IFN-γ^+^ or TNF-α^+^ ATCs, which was near the background level. Another reason for the rather low percentages of ATCs based on the ICS might be the lack of adding professional antigen presenting cells for restimulation. However, our data and those of Papadopoulou et al. indicate that Asp-lysate-expanded ATCs might have a broader reactivity compared to PepMix-expanded ATCs. Furthermore, Asp-lysate and as well as PepMix-expanded ATCs showed high cytotoxic potential was indicated by high killing efficiency and high secretion of cytotoxic mediators upon target recognition. Host response against IFDs after HSCT is mainly driven by a strong Th1 response also known to correlate with a favorable outcome (55), whereas Th2 responses might benefit fungal persistence and infections (12). Indeed, Asp-lysate as well as PepMix-expanded ATCs using our protocol resulted in a dominant proportion of Th1 cells and low proportion of Th2 cells. In addition, both cell products, Asp-lysate- and PepMix-expanded seATCs comprised mainly of polyfunctional (IFN-γ^+^ and TNF-α^+^) CD4^+^ helper T cells but also CD8^+^ cytotoxic T cells (CTLs), both with high proportions of T_CM_ and T_EM_ cells, thus allowing a fast as well as sustained antifungal activity (41). Our data show that ATCs against Asp-lysate as well as PepMix recognized *Aspergillus fumigatus* and exerted a direct antifungal activity *in vitro* against *A. fumigatus* hyphae. Moreover, we show for the first time that both, the IFN-γ CSA (analog to the clinical-scale IFN-γ CCS) and the STE are suitable approaches to generate Asp-specific ATCs.

In our study, Asp-specific ATCs were directly enriched *via* IFN-γ CSA after short *in vitro* stimulation with either Asp-lysate or PepMix. Although the frequencies of IFN-γ^+^ memory csaATCs were comparable after Asp-lysate and PepMix stimulation (**Figures 2A**, **3A**), significantly more Asp-lysate-specific T cells were detected after enrichment. Previously, we demonstrated that the IFN-γ^+^ CSA is the most suitable small-scale assay for selection of best possible donors with respect to cell numbers and purity in the T-cell product, for which IFN-γ^+^ T-cells frequencies obtained *via* CSA were comparable to those obtained during clinical-grade manufacturing *via* IFN-γ CCS (44, 56). Furthermore, IFN-γ CSA can validly determine ACT frequencies and predict the enrichment efficiency in the production process in contrast to other assays like IFN-γ EliSpot assay or ICS. In line with that, our data indicate that pre-screening of donors for precursor frequencies of memory ATCs is not sufficient enough in the donor pre-selection process. Verified enrichment efficiencies in response to the different antigens (Asp-lysate and PepMix) clearly suggesting that pre-testing of potential donors *via* the small-scale IFN-γ CSA is essential for the direct isolation of clinically-grade T-cell products *via* IFN-γ^+^ CCS in high quantity and quality.

So far, adoptive transfer of Asp-specific T cells manufactured by either IFN-γ CCS and Asp-lysates or STE and Asp-derived peptides was demonstrated to be a safe and feasible anti-fungal therapeutic approach in immunocompromised patients. Our study indicates the suitability of both methods for the generation of clinical-grade ATCs using the same HDV starting material. The combination of both manufacturing techniques allows the urgent treatment of patients from the early onset of IFD using the short-term IFN-γ CCS procedure to bridge the following 2-3 weeks until infusion of seATCs including high proportions of central and effector memory CD4^+^ and CD8^+^ T cells which will occur from the same donor material. Furthermore, our procedures could be easily implemented under GMP-compliant conditions allowing the immediate and sustained treatment of patients suffering from infections with Asp. Prospective clinical trials are needed to prove the feasibility, safety, and efficacy of this promising cellular treatment approach.

## Data availability statement

The original contributions presented in the study are included in the article/**Supplementary Material**. Further inquiries can be directed to the corresponding author.

## Ethics statement

The studies involving human participants were reviewed and approved by Hannover Medical School. The patients/participants provided their written informed consent to participate in this study.

## Author contributions

Conceived and designed the experiments: RG, BE-V. Performed the experiments: ST-Z, NF, CH-F, JS, TB. Analyzed the data: RG, ST-Z, BE-V, ES, NF, CH-F, JS, TB. Wrote the paper: RG, ES, BE-V, ST-Z. Critically revised the manuscript: RG, ES, BE-V, ST-Z, WP, BM-K, RB, VW, TL. Ethic application: RG, VW, BE-V, BM-K. Approved the version to be published: RG, ES, NF, JS, BE-V, ST-Z, WP, BM-K, RB, VW, TL, TB. All authors contributed to the article and approved the submitted version.
