# Supplementary material for: Rapid and sustained T cell-based immunotherapy against invasive fungal disease via a combined two step procedure
Source: Front Immunol. 2023 Apr 5;14:988947. doi: 10.3389/fimmu.2023.988947 (PMC10114046; doi:10.3389/fimmu.2023.988947)
Supplement: Supplementary file 1 [file DataSheet_1.pdf]

Supplementary Material

Supplementary Figures

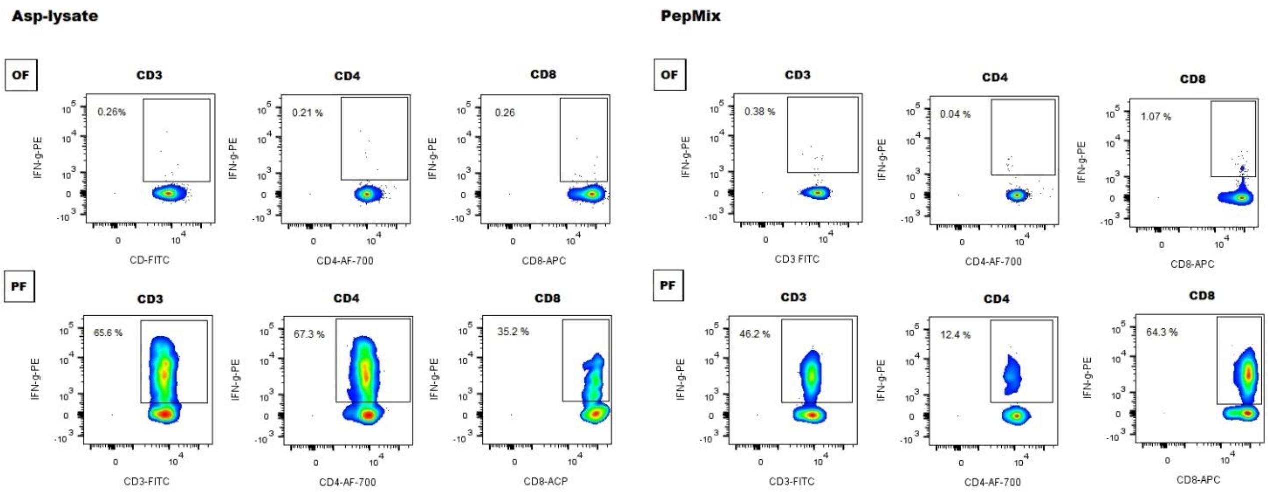

Supplementary Figure 1: Representative result for direct-magnetically enrichment of ACTs via IFN-γ CSA.

The results of the IFN-γ CSA for the representative analysis of aliquots of the respective cell fractions collected before (original fraction, Origin) and after enrichment (positive fraction, PF) were used for detailed characterization of activated IFN-γ^+ T-cell subsets by multicolor flow cytometry. **(A)** Frequency of IFN-γ^+ Asp-specific $CD3^+$, $CD4^+$ and $CD8^+$ T cells stimulated with Asp-lysate for 16 hours before (Origin) and after magnetic enrichment (PF). **(B)** Frequency of IFN-γ^+ Asp-specific $CD3^+$, $CD4^+$ and $CD8^+$ T cells stimulated with PepMix for 16 hour before (Origin) and after magnetic enrichment (PF).

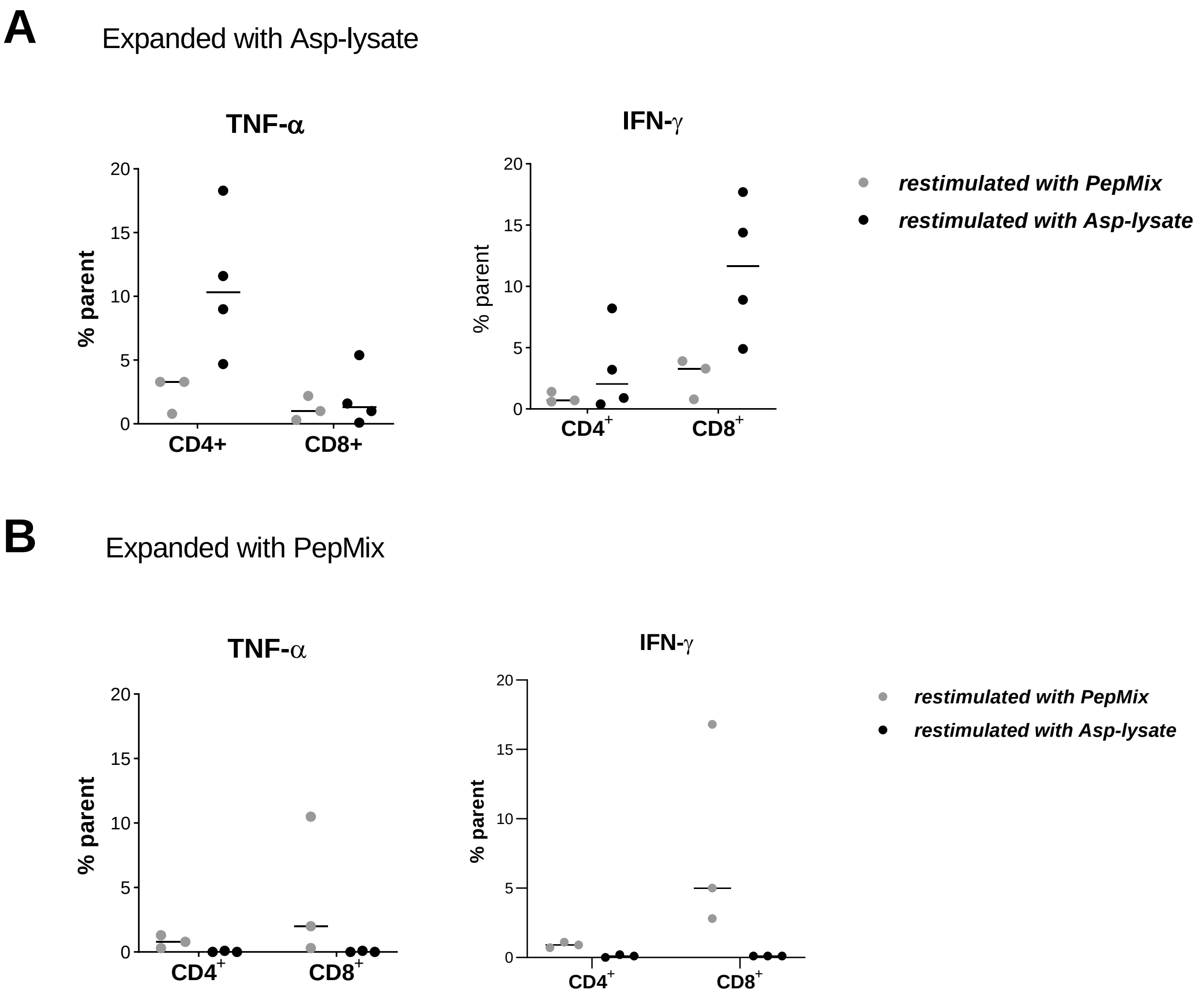

Supplementary Figure 2:

Relative Proportion of IFN-γ^+ or TNF-α^+ positive T-cell subsets ($CD4^+$ and $CD8^+$ T cells) expanded with either **(A)** Asp-lysate or **(B)** PepMix and re-stimulated with either Asp-lysate (black circles) or PepMix (grey circles). Data are shown in total as individual result and mean (n=3-5).

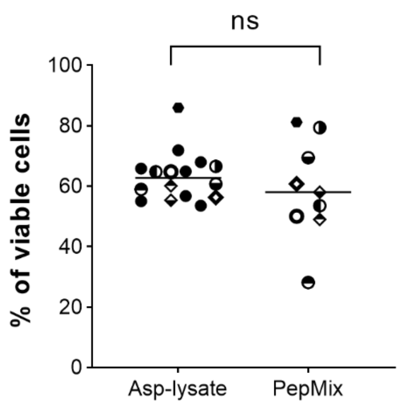

Supplementary Figure 3. Viability assessment of cells after expansion.

Percentage viability shown following expansion with either PepMix or Asp-lysate. Same symbols refer to the same donors; no significant difference between viability following expansion could be detected.

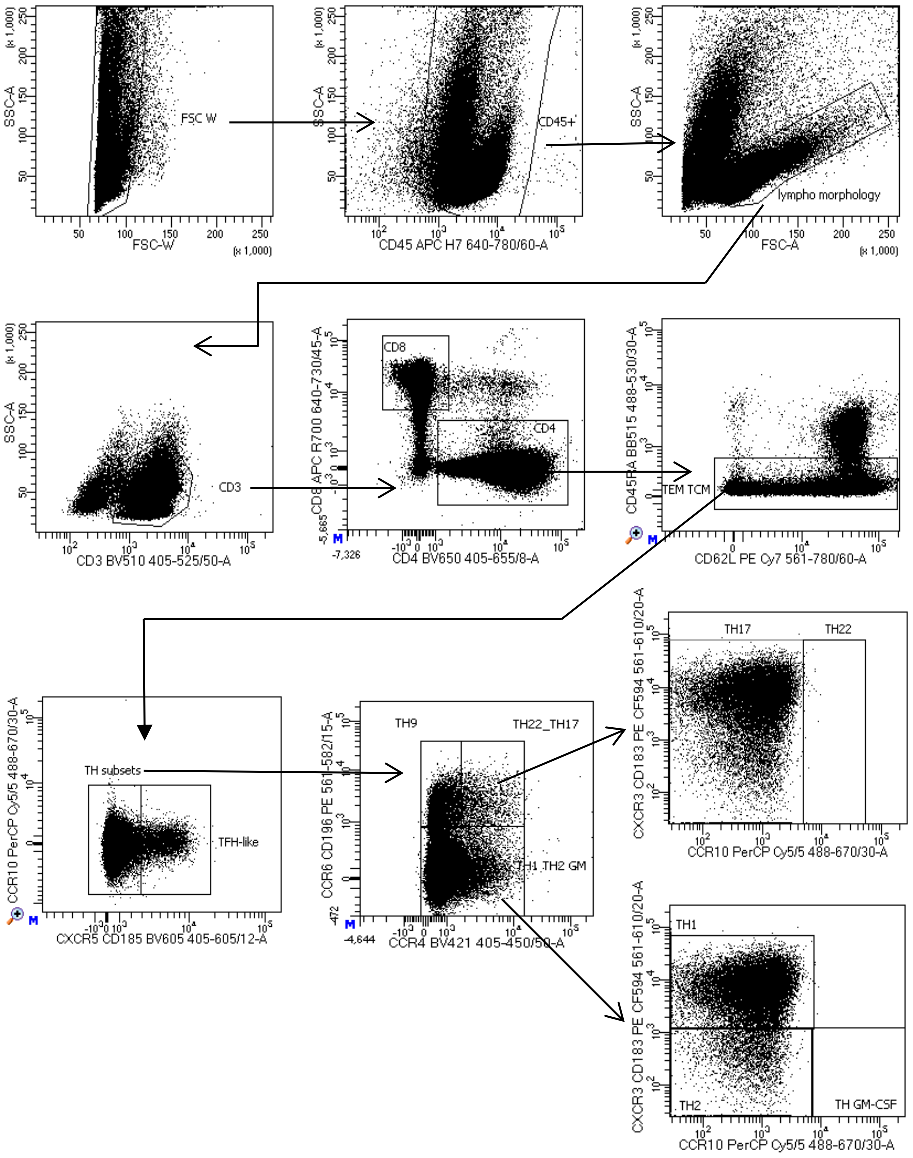

Supplementary Figure 4 Example gating strategy for the identification of helper T-cell subsets

The appropriate gating strategy is indicated by arrows.
